# Time to Change Dosing of Inactivated Quadrivalent Influenza Vaccine in Young Children: Evidence From a Phase III, Randomized, Controlled Trial

**DOI:** 10.1093/jpids/piw068

**Published:** 2017-01-06

**Authors:** Varsha K. Jain, Joseph B. Domachowske, Long Wang, Opokua Ofori-Anyinam, Miguel A. Rodríguez-Weber, Michael L. Leonardi, Nicola P. Klein, Gary Schlichter, Robert Jeanfreau, Byron L. Haney, Laurence Chu, Jo-Ann S. Harris, Kwabena O. Sarpong, Amanda C. Micucio, Jyoti Soni, Vijayalakshmi Chandrasekaran, Ping Li, Bruce L. Innis

**Affiliations:** 1 GSK Vaccines, King of Prussia, Pennsylvania;; 2 SUNY Upstate Medical University, Syracuse, New York;; 3 GSK Vaccines, Wavre, Belgium;; 4 Instituto Nacional de Pediatría de México, Mexico City;; 5 Palmetto Pediatrics, Charleston, South Carolina;; 6 Kaiser Permanente Vaccine Study Center, Oakland, California;; 7 Jean Brown Research, Salt Lake City, Utah;; 8 MedPharmics, Metairie, Louisiana;; 9 Family Health Care of Ellensburg, Ellensburg and Pacific Northwest University, Yakima, Washington;; 10 Benchmark Research, Austin, Texas;; 11 Stormont Vail Health, Topeka, Kansas;; 12 Sealy Center for Vaccine Development University of Texas Medical Branch, Galveston;; 13 Sidney Kimmel Medical College of Thomas Jefferson University, Philadelphia, Pennsylvania;; 14 GlaxoSmithKline Pharmaceuticals Ltd, Bangalore, India

**Keywords:** children, double-dose, inactivated quadrivalent influenza vaccine.

## Abstract

**Background.:**

Children under 3 years of age may benefit from a double-dose of inactivated quadrivalent influenza vaccine (IIV4) instead of the standard-dose.

**Methods.:**

We compared the only United States-licensed standard-dose IIV4 (0.25 mL, 7.5 µg hemagglutinin per influenza strain) versus double-dose IIV4 manufactured by a different process (0.5 mL, 15 µg per strain) in a phase III, randomized, observer-blind trial in children 6–35 months of age (NCT02242643). The primary objective was to demonstrate immunogenic noninferiority of the double-dose for all vaccine strains 28 days after last vaccination. Immunogenic superiority of the double-dose was evaluated post hoc. Immunogenicity was assessed in the per-protocol cohort (N = 2041), and safety was assessed in the intent-to-treat cohort (N = 2424).

**Results.:**

Immunogenic noninferiority of double-dose versus standard-dose IIV4 was demonstrated in terms of geometric mean titer (GMT) ratio and seroconversion rate difference. Superior immunogenicity against both vaccine B strains was observed with double-dose IIV4 in children 6–17 months of age (GMT ratio = 1.89, 95% confidence interval [CI] = 1.64–2.17, B/Yamagata; GMT ratio = 2.13, 95% CI = 1.82–2.50, B/Victoria) and in unprimed children of any age (GMT ratio = 1.85, 95% CI = 1.59–2.13, B/Yamagata; GMT ratio = 2.04, 95% CI = 1.79–2.33, B/Victoria). Safety and reactogenicity, including fever, were similar despite the higher antigen content and volume of the double-dose IIV4. There were no attributable serious adverse events.

**Conclusions.:**

Double-dose IIV4 may improve protection against influenza B in some young children and simplifies annual influenza vaccination by allowing the same vaccine dose to be used for all eligible children and adults.

Influenza has a high incidence and burden in children [1–4]. In particular, influenza B is reported to cause a disproportionate number of influenza-related deaths in children [5]. Routine vaccination of children against influenza is recommended in the United States [6] and other countries. Quadrivalent influenza vaccines containing 2 influenza A strains and 2 influenza B strains are increasingly used in vaccination programs to replace trivalent vaccines.

Inactivated influenza vaccines (IIVs) are administered to adults and children from 3 years of age at a dose of 0.5 mL, containing 15 µg of hemagglutinin (HA) per virus strain. In children under 3 years of age, the United States-licensed standard-dose is 0.25 mL, containing 7.5 µg of HA per virus strain. Both the 15 µg and 7.5 µg doses are available for this age group in some countries, including Canada, Brazil, Mexico, Finland, and the United Kingdom. The 7.5 µg dose was introduced in the 1970s to reduce reactogenicity, including febrile convulsions, associated with the whole virus vaccines available at the time [7–11]. However, young children mount a variable immune response to the 7.5 µg dose [12–14]. Currently available split virus vaccines are better tolerated than whole virus vaccines [10, 15, 16], questioning the practice of using the 7.5 µg dose with IIVs.

The inactivated quadrivalent influenza vaccine (IIV4) manufactured in Quebec, Canada by GSK Vaccines is licensed at a double-dose (15 µg per antigen) for children from 6 months of age in Canada and Mexico, but it is currently only licensed for children 3 years of age and older in the United States. The only IIV4 licensed for use in children 6–35 months of age in the United States is Sanofi Pasteur’s Fluzone Quadrivalent in a standard-dose (7.5 µg per antigen). No other IIV is approved in the United States in this age group either because immunogenic noninferiority to Fluzone could not be demonstrated [17, 18] or because of excessive reactogenicity [19, 20].

If the double-dose vaccine could be administered in young children without adverse effects on tolerability, this age group may benefit from potentially improved immunogenicity. In this study, we describe a phase III study that compared the safety and immunogenicity of a double-dose IIV4 manufactured by GSK Vaccines with the United States-approved standard-dose IIV4 in children 6–35 months of age.

## METHODS

This was a phase III, randomized, controlled, observer-blind, multicenter trial in children 6–35 months of age (ClinicalTrials.gov Identifier NCT02242643). The trial was approved by independent ethics committees or institutional review boards, conducted in accordance with the Declaration of Helsinki, the International Conference on Harmonisation Good Clinical Practice (ICH-GCP) guidelines, ICH Harmonised Tripartite guideline for pediatric populations, and US regulatory requirements. Parents or legally acceptable representatives provided written informed consent.

### Participants, Vaccines, and Study Design

Children in stable health were recruited in the United States and Mexico during the 2014–15 influenza season (Supplementary Appendix). The double-dose IIV4 (GSK Vaccines, Quebec, Canada) contained 15 μg HA of each of the 4 strains: A/California/7/2009 (A/H1N1), A/Texas/50/2012 (A/H3N2), B/Brisbane/60/2008 (B/Victoria), and B/Massachusetts/2/2012 (B/Yamagata). The standard-dose IIV4 (Fluzone Quadrivalent; Sanofi Pasteur, Swiftwater, PA) contained 7.5 μg of HA of each of the same strains.

Children were randomized 1:1 to double-dose or standard-dose IIV4. Allocation to a study group at the investigator site was performed using an internet-based randomization system (SBIR). The randomization algorithm used a minimization procedure to balance the composition of treatment groups, accounting for age (6–17 and 18–35 months), center, and influenza vaccine priming status. The study aimed to enroll 40%–50% of children in the 6–17 months age group. Children were considered vaccine-primed if they had received 2 or more doses of influenza vaccine since July 1, 2010 or at least 1 dose of the 2013–14 influenza vaccine. Vaccine-primed children received a single dose on day 0. Vaccine-unprimed children received 1 dose on day 0 and another on day 28.

### Study Endpoints

Blood for serologic testing was obtained on days 0 and 28 from primed children and on days 0 and 56 from unprimed children. The following parameters were derived from hemagglutination inhibition (HI) titers: (1) geometric mean titer (GMT), (2) seroconversion rate (SCR), (3) seroprotection rate (SPR), and (4) mean geometric increase (MGI). Seroconversion rate was defined as the percentage of participants with either (1) prevaccination reciprocal HI titer <1:10 and a postvaccination reciprocal titer ≥1:40 or (2) prevaccination reciprocal titer ≥1:10 and at least a 4-fold increase in postvaccination reciprocal titer. Seroprotection rate was defined as the percentage of participants who attained reciprocal HI titers of ≥1:40. Mean geometric increase was defined as the geometric mean of the within-subject ratios of the postvaccination/prevaccination reciprocal HI titer.

Parents recorded solicited injection site and general symptoms on the day of vaccination and for the next 6 days. Spontaneously reported symptoms were recorded until 28 days after vaccination. Serious adverse events (SAEs), potential immune-mediated diseases, and medically attended adverse events were recorded until the final study contact on day 180. Monitoring for febrile seizures was carried out throughout the study.

### Study Objectives

The primary objective was to demonstrate immunogenic noninferiority of the double-dose versus the standard-dose IIV4 28 days after completion of the vaccination course. Noninferiority criteria were met if, for each of the 4 vaccine strains, the upper limit of the 95% confidence interval (CI) of the GMT ratio (standard-dose/double-dose) was ≤1.5 and the upper limit of the 95% CI of the difference in SCR (standard-dose minus double-dose) was ≤10%.

If the primary objective was achieved, the secondary objective was to evaluate whether double-dose IIV4 produced an immune response against each of the vaccine strains that met Center for Biologics Evaluation and Research (CBER) criteria, ie, the lower limit of the 95% CI of the SCR was ≥ 40% and the lower limit of the 95% CI of the SPR was ≥ c>70%. Additional secondary objectives were to (1) evaluate GMT, SPR, SCR, and MGI at 28 days after completion of the vaccination course, (2) describe the safety and reactogenicity of the vaccines, and (3) evaluate the relative risk of fever with double-dose versus standard-dose during the 2-day postvaccination period.

A post hoc evaluation was conducted to compare the immune response of the double-dose versus the standard-dose using CBER criteria conventionally applied to establish vaccine lot-to-lot consistency. Immunogenic superiority of the double-dose was concluded if the lower limit of the 95% CI of the GMT ratio (double-dose/standard-dose) was >1.5 and the lower limit of the 95% CI of the difference in SCR (double-dose minus standard-dose) was >10%.

### Statistics

Enrollment of 1200 children per group (1020 evaluable subjects assuming an attrition rate of 15%) was planned to allow a global statistical power of 99% for the primary objective evaluation. The immunogenicity analysis was based on the per-protocol cohort and the safety analysis was based on the intent-to-treat cohort (Supplementary Appendix). Subgroup analyses according to age and priming status were conducted on the per-protocol cohort.

The overall type I error for the study was 5%. If the primary objective was met, the secondary objective of CBER criteria evaluation was tested to provide supportive evidence of immunogenicity. Calculation of 95% CIs is described in the Supplementary Appendix. The group GMT ratio was computed using an analysis of covariance model on the log-transformed titers. Analyses of immunogenicity excluded participants with missing or nonevaluable measurements at the postvaccination time point. Study power was calculated using PASS 2005 (Supplementary Appendix).

## RESULTS

**Figure 1.**
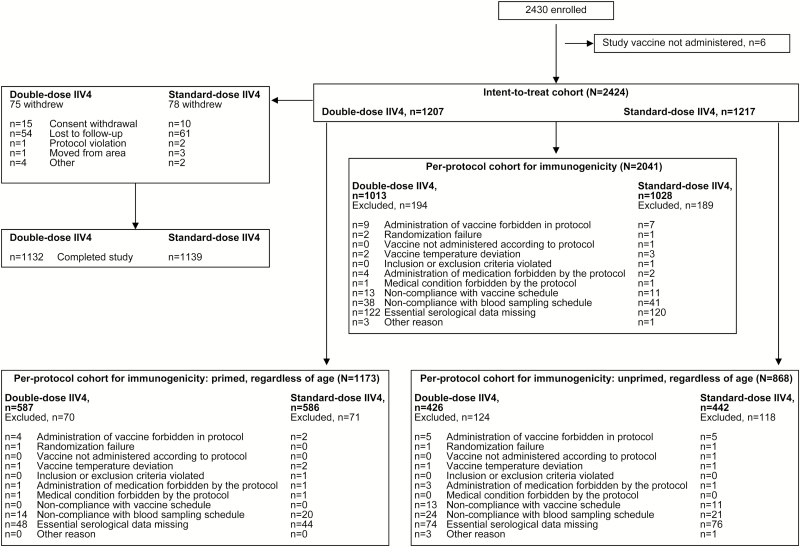
Participant disposition.

A total of 2424 and 2041 children were included in the intent-to-treat cohort and per-protocol cohort, respectively (Figure 1). Demographics were similar in both vaccine groups (Table 1). In the per-protocol cohort, 57.5% of children were vaccine-primed; mean age was 24.6 and 13.3 months for primed and unprimed children, respectively. Other demographic characteristics were similar in primed and unprimed children (Table 1).

**Table 1.** Participant Demographics (Per-Protocol Cohort)

	All Children (Regardless Of Priming Status, 6–35 Months)		Primed Children (6–35 Months)		Unprimed Children (6–35 Months)	
Characteristic	Double-Dose IIV4 N = 1013	Standard-Dose IIV4 N = 1028	Double-Dose IIV4 N = 587	Standard-Dose IIV4 N = 586	Double-Dose IIV4 N = 426	Standard-Dose IIV4 N = 442
Age at first vaccination, months, mean (SD)	19.7 (8.7)	19.9 (8.9)	24.5 (6.2)	24.8 (6.1)	13.1 (7.2)	13.5 (7.9)
Age 6–17 months, n (%)	400 (39.5)	401 (39.0)	74 (12.6)	69 (11.8)	326 (76.5)	332 (75.1)
<12 months, n (%)	213 (21.0)	226 (22.0)	0	0	213 (50.0)	226 (51.1)
Age 18–35 months, n (%)	613 (60.5)	627 (61.0)	513 (87.4)	517 (88.2)	100 (23.5)	110 (24.9)
Female, n (%)	462 (45.6)	496 (48.2)	264 (45.0)	283 (48.3)	198 (46.5)	213 (48.2)
Geographic ancestry, n (%)						
Caucasian/European	647 (63.9)	667 (64.9)	393 (67.0)	400 (68.3)	254 (59.6)	267 (60.4)
African/African American	143 (14.1)	140 (13.6)	89 (15.2)	78 (13.3)	54 (12.7)	62 (14.0)
American Indian or Alaskan Native	23 (2.3)	18 (1.8)	15 (2.6)	13 (2.2)	8 (1.9)	5 (1.1)
South East Asian	17 (1.7)	20 (1.9)	11 (1.9)	14 (2.4)	6 (1.4)	6 (1.4)
Other	183 (18.1)	183 (17.8)	79 (13.5)	81 (13.8)	104 (24.4)	102 (23.1)

Abbreviatioins: IIV4, inactivated quadrivalent influenza vaccine; N, number of participants included in analysis; n, number of participants in stated category; SD, standard deviation.

### Immunogenicity

**Figure 2.**
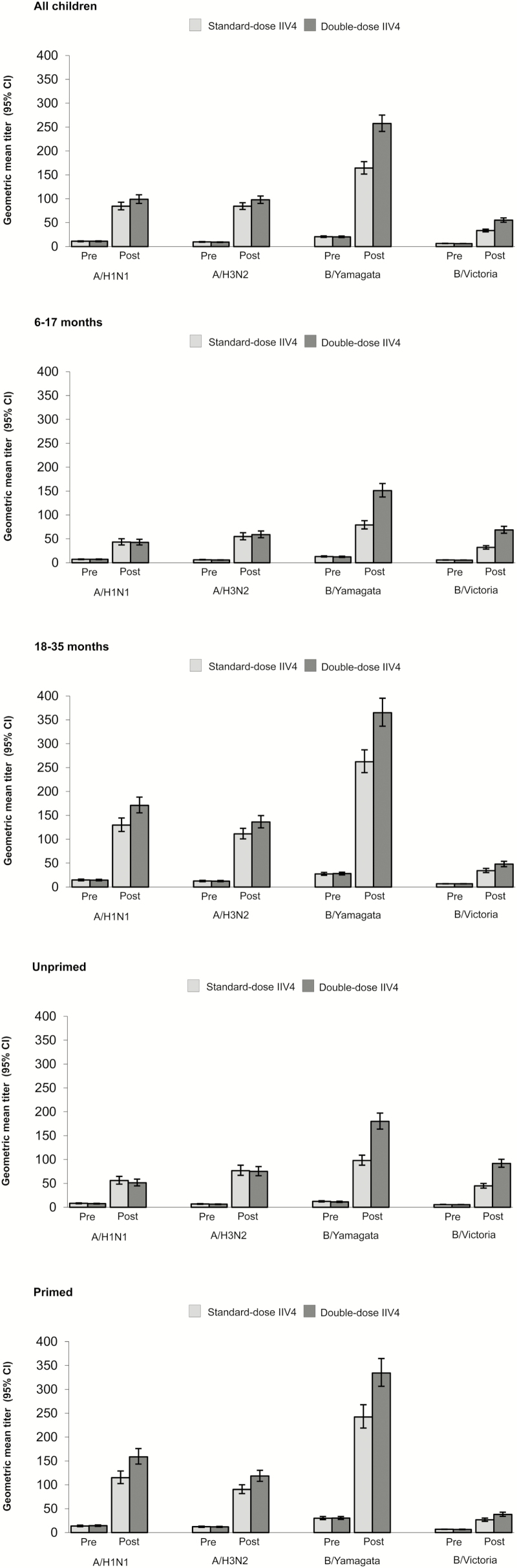
Geometric mean titer for all vaccine strains in all children 6–35 months of age regardless of priming status and in each subgroup prevaccination and 28 days after completion of vaccination series (per-protocol cohort). CI, confidence interval; IIV4, inactivated quadrivalent influenza vaccine.

**Figure 3.**
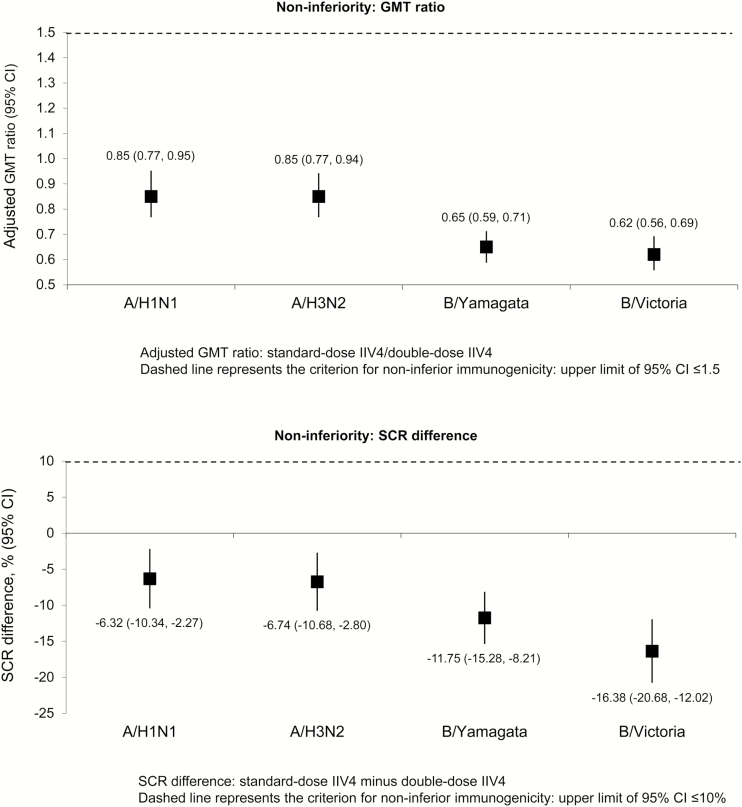
Noninferiority of the double-dose versus the standard-dose in all children 6–35 months of age regardless of priming status: geometric mean titer (GMT) ratio and difference in seroconversion rate (SCR) at 28 days after completion of vaccination series (per-protocol cohort). CI, confidence interval; IIV4, inactivated quadrivalent influenza vaccine.

Both vaccines were immunogenic against all vaccine strains in terms of GMT values (Figure 2). Immunogenic noninferiority of the double-dose IIV4 versus the standard-dose IIV4 was demonstrated for all vaccine strains (Figure 3). Seroconversion rate, SPR, and MGI values were higher in the double-dose group compared with the standard-dose group in the whole study population (6–35 months of age, regardless of priming status; Table 2). The lower limit of the 95% CI for SCR was ≥40% for the double-dose IIV4 against all vaccine strains (Table 2), meeting CBER criteria for demonstration of adequate immunogenicity. For SPR, the lower limit of the 95% CI was ≥70% for all strains except B/Victoria (Table 2).

**Table 2.** Immunogenicity Against Each Vaccine Strain at 28 Days After Completion of Vaccination Series in All Children 6–35 Months of Age Regardless of Priming Status (Per-Protocol Cohort)

	A/H1N1		A/H3N2		B/Yamagata		B/Victoria	
Endpoint	N	Value	N	Value	N	Value	N	Value
GMT, 1/DIL (95% CI)								
Double-dose	1013	98.8 (90.3–108.2)	1013	97.7 (90.3–105.7)	1013	257.5 (240.9–275.3)	1013	55.1 (50.8–59.8)
Standard-dose	1028	84.4 (76.9–92.6)	1028	84.3 (77.6–91.6)	1028	164.2 (151.8–177.6)	1028	33.4 (30.6–36.4)
SCR, % (95% CI)								
Double-dose	972	73.7 (70.8–76.4)	972	76.1 (73.3–78.8)	974	85.5 (83.2–87.7)	973	64.9 (61.8–67.9)
Standard-dose	980	67.3 (64.3–70.3)	980	69.4 (66.4–72.3)	980	73.8 (70.9–76.5)	980	48.5 (45.3–51.6)
SPR, % (95% CI)								
Double-dose	1013	80.4 (77.8–82.8)	1013	82.2 (79.7–84.5)	1013	97.0 (95.8–98.0)	1013	66.0 (63.0–69.0)
Standard-dose	1028	75.4 (72.6–78.0)	1028	77.8 (75.2–80.3)	1028	88.6 (86.5–90.5)	1028	49.8 (46.7–52.9)
MGI (95% CI)								
Double-dose	972	9.0 (8.4–9.7)	972	10.7 (10.0–11.6)	974	12.7 (11.7–13.7)	973	8.7 (8.1–9.4)
Standard-dose	980	7.7 (7.1–8.3)	980	8.9 (8.2–9.7)	980	8.1 (7.5–8.8)	980	5.4 (5.0–5.8)

Abbreviations: CI, confidence interval; DIL, dilution; GMT, geometric mean titer; MGI, mean geometric increase; N, number of participants included in analysis; SCR, seroconversion rate; SPR, seroprotection rate.

**Figure 4.**
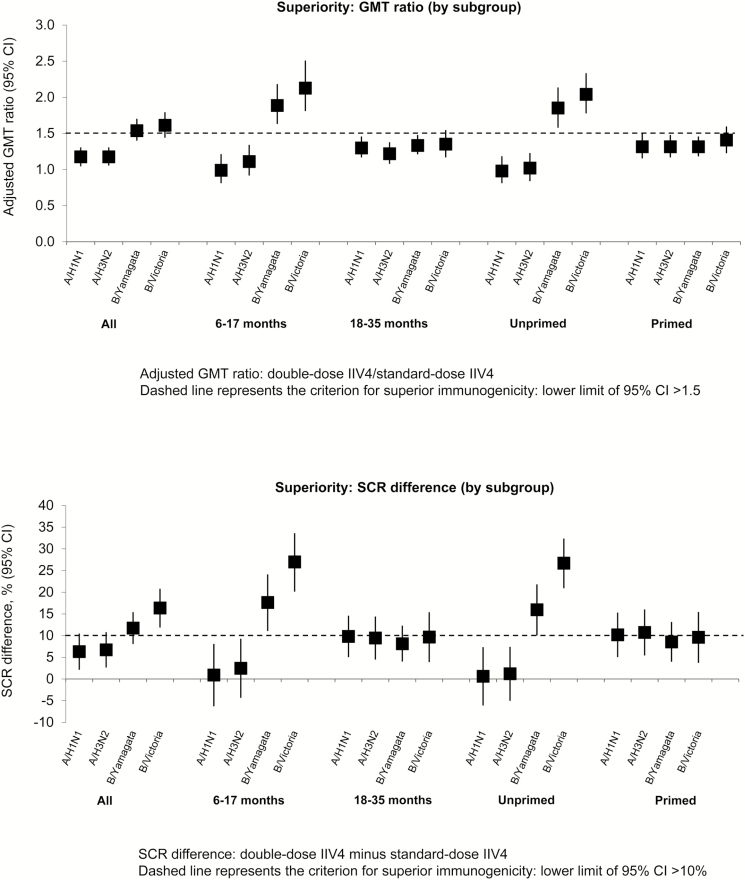
Comparison of immunogenicity of the double-dose versus the standard-dose in all children 6–35 months of age regardless of priming status and in each subgroup: geometric mean titer (GMT) ratio and difference in seroconversion rate (SCR) at 28 days after completion of vaccination series (per-protocol cohort). CI, confidence interval; IIV4, inactivated quadrivalent influenza vaccine.

Immunogenicity was higher in the double-dose group compared with the standard-dose group, particularly against vaccine B strains in children 6–17 months of age and unprimed children (Table 3; Figure 2). When the unprimed group was further evaluated by age, it could be seen that the main difference between vaccines occurred in children 6–17 months of age. These observations prompted us to perform the post hoc evaluation comparing the immune response elicited by the vaccines in the whole study population and according to age group and priming status. The analysis indicated superior immunogenicity of the double-dose IIV4 against both vaccine B strains in children 6–17 months of age and all unprimed children. In children 6–17 months of age, the GMT ratio was 1.89 (95% CI, 1.64–2.17) for B/Yamagata and 2.13 (95% CI, 1.82–2.50) for B/Victoria (Figure 4 and Supplementary Table 1). Corresponding values in all unprimed children were 1.85 (95% CI, 1.59–2.13) and 2.04 (95% CI, 1.79–2.33). Superior immunogenicity of the double-dose was also observed for the same groups in terms of SCR difference (Figure 4 and Supplementary Table 1).

**Table 3.** Comparison of Immunogenicity of the Double-Dose Versus the Standard-Dose According to Age and Priming Status at 28 Days After Completion of Vaccination Series (Per-Protocol Cohort)

	A/H1N1		A/H3N2		B/Yamagata		B/Victoria	
Endpoint	N	Value	N	Value	N	Value	N	Value
6–17 months (regardless of priming status)								
GMT, 1/DIL (95% CI)								
Double-dose	400	42.7 (37.1–49.0)	400	58.9 (52.2–66.4)	400	151.0 (137.4–165.9)	400	68.7 (61.8–76.3)
Standard-dose	401	43.2 (37.3–50.0)	401	54.8 (47.9–62.7)	401	79.1 (70.9–88.1)	401	31.9 (28.4–35.7)
SPR, % (95% CI)								
Double dose	400	61.3 (56.3–66.1)	400	70.3 (65.5–74.7)	400	94.3 (91.5–96.3)	400	78.3 (73.9–82.2)
Standard-dose	401	59.9 (54.9–64.7)	401	67.8 (63.0–72.4)	401	77.6 (73.2–81.5)	401	51.4 (46.4–56.4)
SCR, % (95% CI)								
Double-dose	376	58.5 (53.3–63.5)	376	69.1 (64.2–73.8)	376	79.5 (75.1–83.5)	376	77.4 (72.8–81.5)
Standard-dose	375	57.6 (52.4–62.7)	375	66.7 (61.6–71.4)	375	61.9 (56.7–66.8)	375	50.4 (45.2–55.6)
MGI (95% CI)								
Double-dose	376	6.0 (5.3–6.8)	376	10.2 (9.0–11.6)	376	12.3 (10.7–14.3)	376	12.3 (11.0–13.8)
Standard-dose	375	6.1 (5.2–7.1)	375	8.8 (7.7–10.2)	375	6.1 (5.3–7.0)	375	5.7 (5.1–6.4)
18–35 months (regardless of priming status)								
GMT, 1/DIL (95% CI)								
Double-dose	613	170.9 (155.2–188.3)	613	136.0 (123.7–149.6)	613	364.8 (336.7–395.3)	613	47.8 (42.6–53.6)
Standard-dose	627	129.6 (116.3–144.3)	627	111.1 (100.6–122.7)	627	262.1 (239.3–287.1)	627	34.4 (30.4–38.8)
SPR, % (95% CI)								
Double-dose	613	92.8 (90.5–94.7)	613	90.0 (87.4–92.3)	613	98.9 (97.7–99.5)	613	58.1 (54.1–62.0)
Standard-dose	627	85.3 (82.3–88.0)	627	84.2 (81.1–87.0)	627	95.7 (93.8–97.1)	627	48.8 (44.8–52.8)
SCR, % (95% CI)								
Double-dose	596	83.2 (80.0–86.1)	596	80.5 (77.1–83.6)	598	89.3 (86.5–91.7)	597	57.0 (52.9–61.0)
Standard-dose	605	73.4 (69.7–76.9)	605	71.1 (67.3–74.7)	605	81.2 (77.8–84.2)	605	47.3 (43.2–51.3)
MGI (95% CI)								
Double-dose	596	11.7 (10.7–12.8)	596	11.1 (10.1–12.1)	598	12.9 (11.8–14.0)	597	7.0 (6.4–7.7)
Standard-dose	605	8.9 (8.1–9.8)	605	9.0 (8.2–9.9)	605	9.7 (8.9–10.6)	605	5.2 (4.7–5.7)
Primed (regardless of age)								
GMT, 1/DIL (95% CI)								
Double-dose	587	158.8 (143.3–176.0)	587	118.4 (107.5–130.3)	587	334.3 (306.4–364.7)	587	38.1 (34.0–42.8)
Standard-dose	586	115.0 (102.6–128.9)	586	90.4 (81.8–100.0)	586	242.2 (219.1–267.7)	586	26.7 (23.6–30.3)
SPR, % (95% CI)								
Double-dose	587	90.6 (88.0–92.9)	587	87.2 (84.2–89.8)	587	98.1 (96.7–99.1)	587	49.4 (45.3–53.5)
Standard-dose	586	82.1 (78.7–85.1)	586	80.2 (76.7–83.4)	586	93.7 (91.4–95.5)	586	40.1 (36.1–44.2)
SCR, % (95% CI)								
Double-dose	570	80.5 (77.0–83.7)	570	77.9 (74.3–81.2)	572	86.5 (83.5–89.2)	571	48.0 (43.8–52.2)
Standard-dose	563	70.3 (66.4–74.1)	563	67.1 (63.1–71.0)	563	78.0 (74.3–81.3)	563	38.4 (34.3–42.5)
MGI (95% CI)								
Double-dose	570	10.9 (10.0–12.0)	570	10.0 (9.1–10.9)	572	10.7 (9.9–11.6)	571	5.6 (5.1–6.1)
Standard-dose	563	8.5 (7.7–9.3)	563	7.6 (6.9–8.3)	563	8.2 (7.6–8.9)	563	4.0 (3.6–4.4)
Unprimed (regardless of age)								
GMT, 1/DIL (95% CI)								
Double-dose	426	51.4 (44.7–59.1)	426	75.0 (66.0–85.3)	426	179.8 (163.7–197.4)	426	91.7 (83.8–100.3)
Standard-dose	442	56.0 (48.4–64.8)	442	76.8 (66.9–88.3)	442	98.1 (88.1–109.3)	442	44.8 (40.1–50.0)
SPR, % (95% CI)								
Double-dose	426	66.2 (61.5–70.7)	426	75.4 (71.0–79.4)	426	95.5 (93.1–97.3)	426	89.0 (85.6–91.8)
Standard-dose	442	66.5 (61.9–70.9)	442	74.7 (70.3–78.7)	442	81.9 (78.0–85.4)	442	62.7 (58.0–67.2)
SCR, % (95% CI)								
Double-dose	402	63.9 (59.0–68.6)	402	73.6 (69.0–77.9)	402	84.1 (80.1–87.5)	402	88.8 (85.3–91.7)
Standard-dose	417	63.3 (58.5–67.9)	417	72.4 (67.9–76.7)	417	68.1 (63.4–72.6)	417	62.1 (57.3–66.8)
MGI (95% CI)								
Double-dose	402	6.9 (6.1–7.8)	402	11.8 (10.4–13.4)	402	16.0 (13.9–18.5)	402	16.2 (14.8–17.8)
Standard-dose	417	6.8 (5.9–7.8)	417	11.2 (9.7–12.9)	417	8.0 (6.9–9.3)	417	8.0 (7.2–8.8)
Unprimed (6–17 months)								
GMT, 1/DIL (95% CI)								
Double-dose	326	36.2 (31.3–41.9)	326	56.7 (49.7–64.8)	326	146.8 (132.5–162.7)	326	84.6 (76.7–93.3)
Standard-dose	332	38.0 (32.5–44.4)	332	54.1 (46.6–62.7)	332	71.1 (63.6–79.4)	332	35.5 (31.5–40.0)
SPR, % (95% CI)								
Double-dose	326	57.4 (51.8–62.8)	326	68.7 (63.4–73.7)	326	94.2 (91.0–96.5)	326	87.4 (83.3–90.8)
Standard-dose	332	57.2 (51.7–62.6)	332	68.4 (63.1–73.3)	332	75.9 (70.9–80.4)	332	55.1 (49.6–60.6)
SCR, % (95% CI)								
Double-dose	304	54.9 (49.2–60.6)	304	68.4 (62.9–73.6)	304	79.3 (74.3–83.7)	304	87.2 (82.9–90.7)
Standard-dose	309	55.0 (49.3–60.7)	309	68.0 (62.4–73.1)	309	58.9 (53.2–64.4)	309	54.4 (48.6–60.0)
MGI (95% CI)								
Double-dose	304	5.5 (4.7–6.3)	304	10.3 (8.9–11.9)	304	12.8 (10.7–15.1)	304	15.7 (14.0–17.6)
Standard-dose	309	5.5 (4.6–6.5)	309	9.1 (7.7–10.6)	309	5.6 (4.7–6.6)	309	6.5 (5.8–7.3)
Unprimed (18–35 months)								
GMT, 1/DIL (95% CI)								
Double-dose	100	161.7 (125.9–207.7)	100	187.0 (143.6–243.6)	100	347.7 (296.3–407.9)	100	119.2 (96.8–146.7)
Standard-dose	110	180.9 (141.0–232.1)	110	222.0 (173.3–284.4)	110	260.0 (217.5–310.8)	110	90.5 (73.2–111.8)
SPR, % (95% CI)								
Double-dose	100	95.0 (88.7–98.4)	100	97.0 (91.5–99.4)	100	100 (96.4–100)	100	94.0 (87.4–97.8)
Standard-dose	110	94.5 (88.5–98.0)	110	93.6 (87.3–97.4)	110	100 (96.7–100)	110	85.5 (77.5–91.5)
SCR, % (95% CI)								
Double-dose	98	91.8 (84.5–96.4)	98	89.8 (82.0–95.0)	98	99.0 (94.4–100)	98	93.9 (87.1–97.7)
Standard-dose	108	87.0 (79.2–92.7)	108	85.2 (77.1–91.3)	108	94.4 (88.3–97.9)	108	84.3 (76.0–90.6)
MGI (95% CI)								
Double-dose	98	13.9 (11.6–16.8)	98	18.0 (14.1–23.1)	98	32.5 (26.3–40.1)	98	18.0 (15.3–21.2)
Standard-dose	108	12.4 (10.2–15.1)	108	20.7 (15.6–27.4)	108	22.8 (18.4–28.3)	108	14.2 (11.9–16.8)

Abbreviations: CI, confidence interval; DIL, dilution; GMT, geometric mean titer; MGI, mean geometric increase; N, number of participants included in analysis; SCR, seroconversion rate; SPR, seroprotection rate.

### Safety and Reactogenicity

Pain was the most common solicited injection site symptom, occurring in approximately 40% of children in both vaccine groups; severe (grade 3) pain occurred in 2.9% (95% CI, 2.0–4.1) and 1.7% (95% CI, 1.0–2.6) of children with the double-dose and standard-dose, respectively (Table 4). Fever (38.0°C) was reported in approximately 8% of children up to 7 days postvaccination; fever >39.0°C occurred in approximately 2% of children (Table 4). During the 2-day postvaccination period (days 0–1), the incidence of fever (≥38.0°C) was similar in both groups (Table 4), and the relative risk (double-dose/standard-dose) was 0.97 (95% CI, 0.62–1.52; *P* = .9777). Twenty-two SAEs occurred in the double-dose group and 21 in the standard-dose group (Table 4), none considered related to vaccination. Febrile seizure was reported in 5 children in the double-dose group and in 4 children in the standard-dose group (Table 4).

**Table 4.** Safety Outcomes Reported Throughout the Study (Intent-to-Treat Cohort)

	Double-Dose IIV4 N = 1207^a^		Standard-Dose IIV4 N = 1217^a^	
Adverse event	No. Patients With Symptom	% (95% CI)	No. Patients With Symptom	% (95% CI)
Solicited^b^ injection site symptoms during 7-day postvaccination period				
Pain	509	44.0 (41.1–46.9)	462	40.1 (37.3–43.0)
Grade 3^c^	34	2.9 (2.0–4.1)	19	1.7 (1.0–2.6)
Redness	16	1.4 (0.8–2.2)	16	1.4 (0.8–2.2)
Grade 3^c^	0	-	0	-
Swelling	11	1.0 (0.5–1.7)	5	0.4 (0.1–1.0)
Grade 3^c^	0	-	0	-
Solicited general symptoms during 7-day postvaccination period				
Drowsiness	471	40.6 (37.8–43.5)	471	40.9 (38.0–43.8)
Grade 3^c^	36	3.1 (2.2–4.3)	34	3.0 (2.1–4.1)
Fever (≥38.0°C)	91	7.9 (6.4–9.6)	86	7.5 (6.0–9.1)
>39.0°C	25	2.2 (1.4–3.2)	17	1.5 (0.9–2.4)
Irritability/fussiness	630	54.4 (51.4–57.3)	582	50.5 (47.6–53.4)
Grade 3^c^	61	5.3 (4.0–6.7)	45	3.9 (2.9–5.2)
Loss of appetite	391	33.7 (31.0–36.5)	385	33.4 (30.7–36.2)
Grade 3^c^	26	2.2 (1.5–3.3)	19	1.6 (1.0–2.6)
Unsolicited (spontaneously reported) symptoms during 28-day postvaccination period				
All	549	45.5 (42.6–48.3)	537	44.1 (41.3–47.0)
Grade 3^c^	70	5.8 (4.5–7.3)	75	6.2 (4.9–7.7)
Related to vaccine	71	5.9 (4.6–7.4)	71	5.8 (4.6–7.3)
Fever reported during 2-day postvaccination period				
All (≥38.0°C)	42	3.6 (2.6–4.9)	43	3.7 (2.7–5.0)
Febrile seizure^d^ during entire study period				
All	5	0.4 (0.1–1.0)	4	0.3 (0.1–0.8)
Medically attended event^e^ during entire study period				
All	727	60.2 (57.4–63.0)	719	59.1 (56.3–61.9)
Potential immune-mediated disease during entire study period^f^				
All	1^g^	0.1 (0.0–0.5)	1^g^	0.1 (0.0–0.5)
Serious adverse event during entire study period^h^				
All	22	1.8 (1.1–2.7)	21	1.7 (1.1–2.6)

Abbreviations: CI, confidence interval; IIV4, inactivated quadrivalent influenza vaccine; N, number of participants included in analysis.

^a^For solicited injection site and general symptoms, only children for whom diary cards were returned are included (injection site symptoms: N = 1156 for double-dose IIV4 and N = 1151 for standard-dose IIV4; general symptoms: N = 1159 for double-dose IIV4 and N = 1152 for standard-dose IIV4).

^b^All solicited injection-site symptoms were considered related to vaccination.

^c^Grade 3 events were defined as follows: pain: child cried when the limb was moved or the limb was spontaneously painful; redness and swelling: >100 mm surface diameter; drowsiness and irritability/fussiness: prevented normal activity; loss of appetite: did not eat at all; spontaneously reported symptom: prevented normal activity.

^d^In the double-dose group, seizures occurred 5, 50, 88, 106, and 168 days after the first vaccine dose. In the standard-dose group, 1 seizure occurred 178 days after the first vaccine dose and the others 39, 74, and 80 days after the second vaccine dose. All children recovered, and none of the seizures was considered by the investigator to be related to vaccination.

^e^Hospitalization, emergency room visit, medical practitioner visit.

^f^Autoimmune diseases and other inflammatory and/or neurologic disorders that may or may not have an autoimmune etiology, according to a protocol-specified list or investigators’ judgment.

^g^Kawasaki’s disease in the double-dose group and erythema multiforme in the standard-dose group, neither related to vaccination.

^h^Serious adverse events were defined as any untoward medical occurrence that results in death, is life-threatening, requires hospitalization or prolongs hospitalization, or results in disability or incapacity.

There was a modest increase in reactogenicity with regard to general symptoms in children 6–17 months of age compared with those aged 18–35 months with both the double-dose and standard-dose vaccines. With the double-dose vaccine, the fold-difference between the younger and older age groups ranged from 1.3 for loss of appetite to 2.7 for fever ≥38.0°C. With the standard-dose, the fold-difference ranged from 1.2 for loss of appetite to 1.6 for drowsiness and fever ≥38.0°C. The difference between age groups was unlikely to be due to chance because, in general, 95% CIs did not overlap. However, there were overlapping 95% CIs and thus no apparent age group differences with the standard-dose vaccine for fever ≥38.0°C and loss of appetite.

## DISCUSSION

The introduction of IIV4 provides an opportunity to review long-accepted practices in administration of influenza vaccines. Since the 1970s, the standard-dose of IIVs in children less than 3 years of age has been 7.5 µg per antigen, half the dose given to older children and adults. The lower dose was intended to reduce reactogenicity and febrile convulsions observed with the whole virus vaccines that were in use at the time [7–11]. However, young children mount a variable immune response to this lower dose, especially against vaccine B strains [12–14]. In particular, vaccine-naive children less than 3 years of age mount a lower immune response compared with older or vaccine-primed children [14, 21–23]. The immune response in this vulnerable group could be improved by a change in practice to administer the double-dose, ie, same dose as used for children 3 years of age and above, and for adults. Increasing the immunogenicity of IIVs for young children is expected to improve their effectiveness, because the postvaccination HI antibody titer is inversely related to the risk of illness [24, 25]. However, there is controversy regarding the HI antibody titer necessary to offer high-level effectiveness [24, 25].

In the present study, both the double-dose and the standard-dose IIV4s were immunogenic against all vaccine strains in primed and unprimed children 6–35 months of age. The primary objective of the study—to fulfill US licensure criteria by demonstrating immunogenic noninferiority of the investigational IIV4 to a licensed IIV4 and acceptable safety of the investigational IIV4—was achieved. Most children receiving the double-dose IIV4 seroconverted (SCRs, 64.9%–85.5%), and most children achieved seroprotection (SPRs, 66.0%–97.0%). Similar immune responses have been achieved with the double-dose IIV4 in children of the same age in small studies conducted in 3 prior seasons [26–28].

Greater antibody responses were observed with the double-dose IIV4 compared with the standard-dose, prompting us to perform a post hoc analysis to evaluate whether the double-dose elicited a superior immune response in terms of the CBER criteria usually applied to establish lot-to-lot consistency of influenza vaccines. In this analysis, the double-dose IIV4 did not reach superiority to the standard-dose in the overall population, the older age group (18–35 months), or previously primed children. However, in the younger age group (6–17 months) and in all unprimed children, the double-dose IIV4 met the applied superiority immune response criteria compared with the standard-dose against the B strains. It should be noted that the unprimed group was predominantly 6–17 months of age.

Several previous studies have compared the HI antibody response elicited by a double-dose versus a standard-dose IIV. The results of the present large phase III study contrast with those of a phase II study comparing GSK’s double-dose IIV4 with the United States-approved standard-dose inactivated trivalent influenza vaccine (IIV3) (the corresponding IIV3 to the licensed IIV4 comparator used in the present study) [26]. In the phase II study, the immune response with the double-dose and the standard-dose was similar against the strains common to both vaccines, but the sample size was too small to reliably detect differential immunogenicity against the vaccine B strains, especially in children 6–17 months of age [26]. Two other small studies compared the immunogenicity of the United States-approved IIV3 administered as a standard or double-dose to young children in different years, with contrasting results [21, 29]. A 2008–09 trial found that a double-dose IIV3 elicited a higher immune response than a standard-dose in vaccine-unprimed children 6–23 months of age, reaching statistical significance in children 6–11 months of age for 2 of 3 vaccine strains [21]. However, a 2010–12 trial found no difference in immunogenicity between standard-dose versus double-dose IIV3s in unprimed children [29]. This prior experience highlights the necessity for trials of adequate size to reliably establish treatment benefit, and it suggests that observations made in 1 year may not be repeated in other years, because the baseline immunity of young children may vary. Furthermore, the dose effect on immunogenicity among IIVs may differ according to their manufacturing process [23, 26].

In the present study, the double-dose and standard-dose IIV4s had a similar reactogenicity profile despite the higher antigen content and volume of the double-dose. Injection site symptoms, including pain, occurred at a similar rate in both groups. There was no difference in the rate of fever over the 2-day postvaccination period between the 2 groups. Febrile seizures occurred at a similar rate in both groups, none were reported within 2 days of vaccination, and none were considered related to the vaccine. The finding that the higher antigen dose and volume in this study did not adversely affect tolerability in children confirms previous findings from studies comparing reactogenicity and safety of double-dose versus standard-dose IIVs [21–23, 26, 29], and IIV4s versus IIV3s [26, 27, 30–32].

## CONCLUSIONS

In conclusion, a double-dose IIV4 may afford greater protection in young children against influenza B. Increased protection against influenza B, a potentially serious and life-threatening illness particularly in young children [33], would be a beneficial clinical outcome. Use of the same vaccine dose for all eligible ages would also simplify the annual influenza vaccine campaign and reduce cost [34] and logistic complexity. This study provides evidence to support a change in clinical practice to use a double-dose IIV4 (15 µg per antigen) in all children 6 months of age and older, once that dosing for a vaccine product has been approved.

## Supplementary Data

Supplementary materials are available at *Journal of The Pediatric Infectious Diseases Society* online.

## Supplementary Material

Jain_et_al_SUPPLEMENTARY_APPENDIX_revised

## Notes

***Acknowledgments.*** We are indebted to the participating study volunteers and their parents, clinicians, nurses, and laboratory technicians at the study sites. In particular, we thank Christopher Chambers, Larkin Wadsworth, Wendy Daly, William Johnston, Michael Levin, William Douglas, Agnes Schultz, Edward Zissman, Terry Poling, Paul Bernhardson, Brad Brabec, Stephen Russel, and Mary Tipton who provided support and cared for study participants. We thank the teams of GSK Vaccines, in particular, Silvija Jarnjak, Arshad Amanullah, Els Praet, and Rafik Bekkat-Berkani. Finally, we thank Mary L. Greenacre (An Sgriobhadair, UK, on behalf of GSK Vaccines) for providing medical writing services and Bruno Dumont and Véronique Gochet (Business and Decision Life Sciences, on behalf of GSK Vaccines) for editorial assistance and manuscript coordination.

***Author contributions.*** All authors participated in the design or implementation or analysis of the study, interpretation of the study, and the development of this manuscript. All authors had full access to the data and gave final approval before submission.

***Financial support.*** This work was supported by GlaxoSmithKline Biologicals SA. GlaxoSmithKline Biologicals SA paid for all costs associated with the development of this manuscript.

***Potential conflicts of interest.*** V. K. J., L. W., and P. L. were employed by the GSK group of companies at the time of the study. V. K. J. is currently employed by the Bill and Melinda Gates Foundation. L. W. is currently employed by Merck Research Laboratory. P. L. is currently employed by Pfizer. O. O.-A., J. S., V. C., and B. L. I. are currently employed by the GSK group of companies. V. K. J., L. W., O. O.-A., P. L., and B. L. I. currently hold shares in the GSK group of companies. J. B. D. reports payments from the GSK group of companies, during the conduct of the study, and reports grants and others from the GSK group of companies, Pfizer, Sanofi Pasteur, and MedImmune for consultancy services, outside the submitted work. M. L. L. reports payments from the GSK group of companies, during the conduct of the study. N. P. K. reports payments from the GSK group of companies, during the conduct of the study, and grants from Sanofi Pasteur, Novartis, Pfizer, Protein Science, MedImmune, Merck & Co, and Nuron Biotech, outside the submitted work. B. L. H. reports payments and nonfinancial support from the GSK group of companies, during the conduct of the study. J. S. H. reports payments from the GSK group of companies, during the conduct of the study. A. C. M. reports payments from the GSK group of companies, during the conduct of the study.
