# Supplementary material for: Time to Change Dosing of Inactivated Quadrivalent Influenza Vaccine in Young Children: Evidence From a Phase III, Randomized, Controlled Trial
Source: J Pediatric Infect Dis Soc. 2017 Jan 6;6(1):9–19. doi: 10.1093/jpids/piw068 (PMC5907868; doi:10.1093/jpids/piw068)
Supplement: Jain_et_al_SUPPLEMENTARY_APPENDIX_revised [file piw068_suppl_jain_et_al_supplementary_appendix_revised.doc]

# Supplementary Appendix

## Participants and vaccine administration

The health of the children was determined by clinical examination and medical history. Children were included regardless of influenza vaccination history, but could not have received any seasonal or pandemic influenza vaccine within 6 months before the first dose of study vaccine. Children were excluded if they had received immunosuppressant or other immune-modifying drugs for more than 14 days in total within 6 months before the first vaccine dose, immunoglobulins or blood products within 3 months before the first vaccine dose, or any investigational product within 30 days before the first vaccine dose. They were also excluded if they had a history of Guillain-Barré syndrome within six weeks of prior influenza vaccination, allergy to egg or any other vaccine component, a coagulation disorder, an immunosuppressive or immunodeficient condition, or had acute disease and/or temperature ≥38.0°C.

Vaccines were administered intramuscularly in the deltoid muscle of the non-dominant arm for children ≥12 months of age or in the anterolateral region of the left thigh for children <12 months of age.

## Statistics

The primary immunogenicity analysis was based on the per-protocol cohort which included all children who met eligibility criteria, complied with the protocol and their vaccine schedule, and had data available for antibodies against at least one vaccine strain after vaccination. The safety analysis was based on the intent-to-treat cohort which included all vaccinated children for whom safety data were available.

The exact 95% confidence intervals (CI) for a proportion within a group were calculated using Proc StatXact (Clopper & Pearson 1934). Standardized asymptotic 95% CIs for the group difference in proportion were derived from Proc StatXact 7.0 using Newcombe’s method (1998). The 95% CIs for the geometric mean titers (GMTs) were obtained within each group separately. The 95% CIs for the mean of log-transformed antibody titers were first computed assuming that log-transformed values were normally distributed with unknown variance. The 95% CIs for the GMTs were then computed by exponential-transformation of the 95% CIs for the mean of log-transformed titer. The limit of quantitation for the hemagglutination inhibition (HI) assay was 1:10. Samples <1:10 were considered seronegative and given an arbitrary value of 5 for the GMT calculation.

The group GMT ratio was computed using an ANCOVA model on the log-transformed titers. The ANCOVA model included the vaccine group as a fixed effect and the pre-vaccination log10 titer as the regressor. The GMT ratio and 95% CIs were derived by exponential-transformation of the corresponding group contrast in the model. The standardized asymptotic 95% CIs for the difference in SCR were computed using Proc StatXact 7.0.

The 95% CIs for the relative risk of fever between groups were calculated by an exact method conditional to the total number of cases. The p-value was calculated by a 2-sided exact test conditional to the number of cases.

Assuming a GMT ratio of 1.0 and an SCR difference of 0% (under Null), 1020 evaluable children per group were required to achieve a global power of 99.07% to demonstrate non-inferiority for all four vaccine strains with both criteria. With 1020 evaluable children per group, the power to meet Center for Biologics Evaluation and Research (CBER) criteria for SCR for all four vaccine strains was >99.99%. However, the power to meet CBER criteria for SPR after vaccination could not be reliably estimated (range <10% to >99%), as it would be conditioned on the cohort’s pre-vaccination exposure to infection or prior vaccination, particularly with B-Victoria lineage viruses that had limited circulation in the 2 years prior to this study (CDC 2014). Assuming an attrition rate of approximately 15%, it was planned to enroll approximately 1200 children per vaccine group.

## References

Centers for Disease Control and Prevention (CDC). Past weekly surveillance reports. Available at: http://www.cdc.gov/flu/weekly/pastreports.htm. Accessed 8 February 2016.

Clopper CJ, Pearson E. The use of confidence or fiducial limits illustrated in the case of the binomial. Biometrika **1934**; 26:404-13.

Newcombe RG. Interval estimation for the difference between independent proportions: comparison of eleven methods. Stat Med **1998**; 17:873-90.

Supplementary Table 1. Comparison of immunogenicity of the double-dose versus the standard-dose in all children and each subgroup: GMT ratio and difference in SCR at 28 days following completion of vaccination series (per-protocol cohort)

|  | **A/H1N1** | | **A/H3N2** | | **B/Yamagata** | | **B/Victoria** | |
| --- | --- | --- | --- | --- | --- | --- | --- | --- |
|  | **N** | **Value** | **N** | **Value** | **N** | **Value** | **N** | **Value** |
| Adjusted GMT ratio (double-dose/standard-dose) |  |  |  |  |  |  |  |  |
| All children | 972/980 | 1.18 (1.05, 1.30) | 972/980 | 1.18 (1.06, 1.30) | 974/980 | 1.54 (1.41, 1.69) | 973/980 | 1.61 (1.45, 1.79) |
| 6–17 months | 376/375 | 0.99 (0.82, 1.20) | 376/375 | 1.11 (0.93, 1.33) | 376/375 | 1.89 (1.64, 2.17) | 376/375 | 2.13 (1.82, 2.50) |
| 18–35 months | 596/605 | 1.30 (1.18­, 1.45) | 596/605 | 1.22 (1.09, 1.37) | 598/605 | 1.33 (1.22, 1.47) | 597/605 | 1.35 (1.18, 1.54) |
| Unprimed | 402/417 | 0.98 (0.82, 1.18) | 402/417 | 1.02 (0.85, 1.22) | 402/417 | 1.85 (1.59, 2.13) | 402/417 | 2.04 (1.79, 2.33) |
| Primed | 570/563 | 1.32 (1.16, 1.49) | 570/563 | 1.32 (1.18, 1.47) | 572/563 | 1.32 (1.19, 1.45) | 571/563 | 1.41 (1.23, 1.59) |
| SCR difference (double-dose minus standard-dose) |  |  |  |  |  |  |  |  |
| All children | 972/980 | 6.32 (2.27, 10.34) | 972/980 | 6.74 (2.80, 10.68) | 974/980 | 11.75 (8.21, 15.28) | 973/980 | 16.38 (12.02, 20.68) |
| 6–17 months | 376/375 | 0.91 (-6.14, 7.95) | 376/375 | 2.48 (-4.2, 9.15) | 376/375 | 17.65 (11.21, 23.99) | 376/375 | 26.99 (20.29, 33.47) |
| 18–35 months | 596/605 | 9.83 (5.19, 14.46) | 596/605 | 9.46 (4.63, 14.27) | 598/605 | 8.14 (4.16, 12.16) | 597/605 | 9.68 (4.03, 15.26) |
| Unprimed | 402/417 | 0.62 (-5.97, 7.2) | 402/417 | 1.21 (-4.88, 7.28) | 402/417 | 15.97 (10.21, 21.68) | 402/417 | 26.70 (21.07, 32.25) |
| Primed | 570/563 | 10.19 (5.19, 15.17) | 570/563 | 10.75 (5.57, 15.90) | 572/563 | 8.56 (4.14, 13.01) | 571/563 | 9.62 (3.86, 15.32) |

CI: confidence interval; GMT: geometric mean titer; N: number of participants included in analysis; SCR: seroconversion rate
